# *PD-1* (*PDCD1*) Promoter Methylation Is a Prognostic Factor in Patients With Diffuse Lower-Grade Gliomas Harboring Isocitrate Dehydrogenase (*IDH*) Mutations

**DOI:** 10.1016/j.ebiom.2018.01.016

**Published:** 2018-01-31

**Authors:** Lea Kristin Röver, Heidrun Gevensleben, Jörn Dietrich, Friedrich Bootz, Jennifer Landsberg, Diane Goltz, Dimo Dietrich

**Affiliations:** a Department of Otolaryngology, Head and Neck Surgery, University Hospital Bonn, Bonn, Germany; b Institute of Pathology, University Hospital Bonn, Bonn, Germany; c Department of Dermatology, Dermato-Oncology Section, University Hospital Bonn, Bonn, Germany; d Institute of Pathology, University Hospital Cologne, Cologne, Germany

**Keywords:** *PD-1*, *PD-L1*, *CTLA-4*, DNA methylation, Lower-grade glioma, Prognosis

## Abstract

Immune checkpoints are important targets for immunotherapies. However, knowledge on the epigenetic modification of immune checkpoint genes is sparse. In the present study, we investigated promoter methylation of *CTLA4*, *PD-L1*, *PD-L2*, and *PD-1* in diffuse lower-grade gliomas (LGG) harboring isocitrate dehydrogenase (*IDH*) mutations with regard to mRNA expression levels, clinicopathological parameters, previously established methylation subtypes, immune cell infiltrates, and survival in a cohort of 419 patients with *IDH*-mutated LGG provided by The Cancer Genome Atlas.

PD-L1, PD-L2, and CTLA-4 mRNA expression levels showed a significant inverse correlation with promoter methylation (PD-L1: *p* = 0.005; PD-L2: *p* < 0.001; CTLA-4: *p* < 0.001). Furthermore, immune checkpoint methylation was significantly associated with age (*PD-L2*: *p* = 0.003; *PD-1*: *p* = 0.015), molecular alterations, i.e. *MGMT* methylation (*PD-L1*: *p* < 0.001; *PD-L2*: *p* < 0.001), *ATRX* mutations (*PD-L2*: *p* < 0.001, *PD-1*: *p* = 0.001), and *TERT* mutations (*PD-L1*: *p* = 0.035, *PD-L2*: *p* < 0.001, *PD-1*: *p* < 0.001, *CTLA4*: *p* < 0.001) as well as methylation subgroups and immune cell infiltrates. In multivariate Cox proportional hazard analysis, *PD-1* methylation qualified as strong prognostic factor (HR = 0.51 [0.34–0.76], *p* = 0.001).

Our findings suggest an epigenetic regulation of immune checkpoint genes via DNA methylation in LGG. *PD-1* methylation may assist the identification of patients that might benefit from an alternative treatment, particularly in the context of emerging immunotherapies.

## Highlights

• *PD-L1*, *PD-L2*, and *CTLA4* DNA methylation in lower-grade gliomas is inversely correlated with mRNA expression levels.
• *PD-1*, *PD-L1*, *PD-L2*, and *CTLA4* DNA methylation in lower-grade gliomas is associated with age and mutation status.
• *PD-1* methylation in lower-grade gliomas is correlated with infiltrating immune cells (B, CD8^+^ T, and dendritic cells).
• *PD-1* methylation is an independent prognostic biomarker for overall survival in lower-grade glioma patients.

Immune checkpoint inhibitors have exhibited dramatic antitumor activity in various malignancies; however, little is known about the epigenetic regulation of immune checkpoint genes in tumors of the central nervous system. In the present study, PD-L1, PD-L2, and CTLA-4 mRNA expression was found to correlate inversely with promoter methylation. Furthermore, *PD-1* methylation was prognostic for survival in lower-grade gliomas harboring isocitrate dehydrogenase (*IDH*) mutations supporting the pivotal role of DNA methylation in the regulation of immune checkpoint genes in gliomas. Prognostic biomarkers may assist the identification of patients that might benefit from an alternative treatment, particularly in the context of immunotherapies.

## 1 Introduction

Gliomas are the most common primary brain tumors accounting for approximately 80% of all brain malignancies in the United States (Ostrom et al., 2016). Diffuse lower-grade gliomas (LGG) often present with very variable clinical appearances and survival rates before fatally progressing to glioblastoma multiforme (Cancer Genome Atlas Research Network et al., 2015). Recent developments in genomic profiling have led to a paradigm shift in the classification of gliomas. As a consequence, the 2016 World Health Organization (WHO) classification includes the molecular characterization of primary brain tumors (e.g. isocitrate dehydrogenase (*IDH*) mutations and codeletions of chromosome arms 1p and 19q (1p/19q co-deletion)) (summarized by Louis et al., 2016). Although the implementation of genetic signatures has led to a better understanding of underlying molecular pathways and more reliable diagnostic criteria, these findings do not fully explain why some LGG patients have far worse courses of disease than others. Recent evidence suggests that DNA methylation profiles might shed light on significantly differing outcomes. Unsupervised cluster analysis of 1122 grade II-III-IV gliomas from The Cancer Genome Atlas (TCGA) identified six methylation groups (LGm1–6) that were in part associated with *IDH* status and further discovered an epigenetic signature that segregated a subgroup of *IDH*-mutant diffuse lower-grade gliomas with unfavorable clinical outcome (Ceccarelli et al., 2016). Mutations in the *IDH1* and *IDH2* genes have previously been identified to lead to a downstream neomorphic enzymatic activity and an accumulation of the onco-metabolite D-2-hydroxyglutarate (D-2HG) in *IDH*-mutant cells (Dang et al., 2009). As D-2HG inhibits key enzymes involved in histone- and DNA-demethylation, excess D-2HG results in DNA hypermethylation. Gliomas harboring *IDH* mutations consequently display a CpG island methylator phenotype (G-CIMP), which is characterized by DNA hypermethylation in CpG-rich domains (Turcan et al., 2012) and has been shown to constitute a subset of tumors with a distinct biology and clinical behavior (Noushmehr et al., 2010). These findings emphasize the relevance of epigenetic alterations as an underlying and therapeutically relevant mechanism in glioma.

Gliomas have long been recognized to induce local and systemic immunosuppression, thereby limiting the innate defense against tumor growth (Gousias et al., 2010). Currently emerging immunomodulatory therapies have therefore generated an increasing interest in these novel therapies as potential treatment options for gliomas. Particularly treatments targeting the immune checkpoints programmed cell death 1 receptor (PD-1)/PD-1 ligand 1 (PD-L1) pathway and cytotoxic T-lymphocyte associated protein 4 (CTLA-4) have exhibited dramatic antitumor efficacy in various tumor entities (Chan et al., 2015, Brahmer et al., 2012, Brahmer et al., 2015, Topalian et al., 2012, Hamid et al., 2013, Wolchok et al., 2013, Larkin et al., 2015, Margolin et al., 2012, Berger et al., 2008, Ribas et al., 2016, Garon et al., 2015). Several clinical trials are currently ongoing to determine the potential of PD-1/PD-L1 and CTLA-4 targeted therapies in high-grade gliomas yielding conflicting results (Omuro et al., 2017, Reardon et al., 2016). Furthermore, several studies have been conducted to determine the prognostic value of PD-L1 in gliomas; however, the results so far have been inconsistent (Xue et al., 2017). The regulation of immune checkpoint genes in glioma, particularly on the epigenetic level, seems to be complex and is only poorly understood. Elucidating the regulatory machinery of immune checkpoints might help to improve patient's treatment, particularly in the view of emerging immunotherapeutic strategies. Recently, inverse correlations between immune checkpoint mRNA levels and promoter methylation indicative of an epigenetic regulation as well as significant associations of immune checkpoint methylation levels with survival have been reported for several hematopoietic and solid neoplasms including acute myeloid leukemia (AML), prostate cancer, colorectal adenocarcinomas, and head and neck squamous cell carcinomas (HNSCC) (Franzen et al., 2018, Gevensleben et al., 2016, Goltz et al., 2016a, Goltz et al., 2016b, Goltz et al., 2017a, Goltz et al., 2017b). However, epigenetic association studies regarding tumors of the central nervous system are lacking so far.

In the present study, we investigated DNA promoter methylation of the immune checkpoints genes *PD-1* (Human Genome Organisation (HUGO) gene symbol: *PDCD1*), *PD-L1* (*CD274*), *PD-L2* (*PDCD1LG2*), and *CTLA-4* (*CTLA4*) in patients with LGG harboring *IDH* mutations with regard to mRNA expression, clinicopathological parameters, previously established methylation subtypes, immune cell infiltrates, and survival.

## 2 Materials and Methods

### 2.1 Patients and Clinical Endpoints

The results shown are entirely based on gene methylation data created by the TCGA Research Network (http://cancergenome.nih.gov/). The cohort comprised fresh-frozen tissues from 419 patients with histologically confirmed LGG from several international centres involved in the TCGA project. Clinical, cytological, and mutational data were obtained from the TCGA Research Network. Additional information on methylation subtypes was taken from Ceccarelli et al. (2016). Patients' characteristics are described in detail in Table 1. Overall survival (OS) was defined as time to death or last follow-up. The mean OS was 24.81 months. The TCGA Research Network acquired written informed consent from all participants. All experiments were carried out according to the World Medical Association Declaration of Helsinki.

**Table 1:** Association of clinicopathological parameters with *PD-L1*, *PD-L2*, *PD-1*, and *CTLA4* promoter methylation in diffuse lower-grade glioma patients (*n* = 419).

Variable	All patients	[%]	Mean *PD-L1* methylation [%]	*p*-Value	Mean *PD-L2* methylation [%]	*p*-Value	Mean *PD-1* methylation [%]	*p*-Value	Mean *CTLA4* methylation [%]	*p*-Value
All patients	419	100.0	36.11		64.49		47.61		91.97	
Gender										
Male	231	55.1	36.56	0.31^c^	64.91	0.57^c^	48.8	0.081^c^	92.31	0.24^c^
Female	187	44.6	35.57		63.88		46.53		91.55	
Unknown	1	0.2								
Age [years]										
Mean	40.87									
Median	39									
≤ 41 years	245	58.5	35.95	0.94^c^	62.75	0.003^c^	49.71	0.015^c^	91.87	0.43^c^
> 41 years	173	41.3	36.36		66.87		45.05		92.12	
WHO classification (2016)										
*IDH*-mut, 1p/19q-codel	169	40.3	36.43	0.44^c^	74.89	< 0.001^c^	44.76	0.002^c^	92.52	0.028^c^
*IDH*-mut, 1p/19q-non-codel	250	59.7	35.9		57.46		49.96		91.61	
Methylation subgroups^a^										
LGm1	45	10.7	30.59	< 0.001^b^	50.57	< 0.001^b^	40.48	0.001^b^	88.18	< 0.001^b^
LGm2	251	59.9	36.72		60.82		50.06		92.32	
LGm3	123	29.4	36.9		77.06		46.07		92.66	
*MGMT* promoter status^a^										
Methylated	389	92.84	36.43	< 0.001^c^	65.33	< 0.001^c^	47.44	0.068^c^	92.12	0.11^c^
Unmethylated	30	7.2	31.9		53.58		53.29		90.06	
*ATRX* status										
Mutant	181	43.2	36.19	0.79	57.86	< 0.001^c^	51.15	0.001^c^	91.71	0.63^c^
Wildtype	238	56.8	36.05		69.53		45.36		92.17	
*TERT* promoter status										
Mutant	93	22.2	36.94	0.035^c^	73.53	< 0.001^c^	45.47	< 0.001^c^	93.22	< 0.001^c^
Wildtype	143	34.1	36.69		58.19		52.6		92.45	
Unknown	183	43.7								

a Data taken from Ceccarelli et al. (2016).

b Data taken from Kruskal-Wallis test.

c Data taken from Wilcoxon-Mann-Whitney test.

### 2.2 Promoter Methylation Analyses

**Fig. 1:**
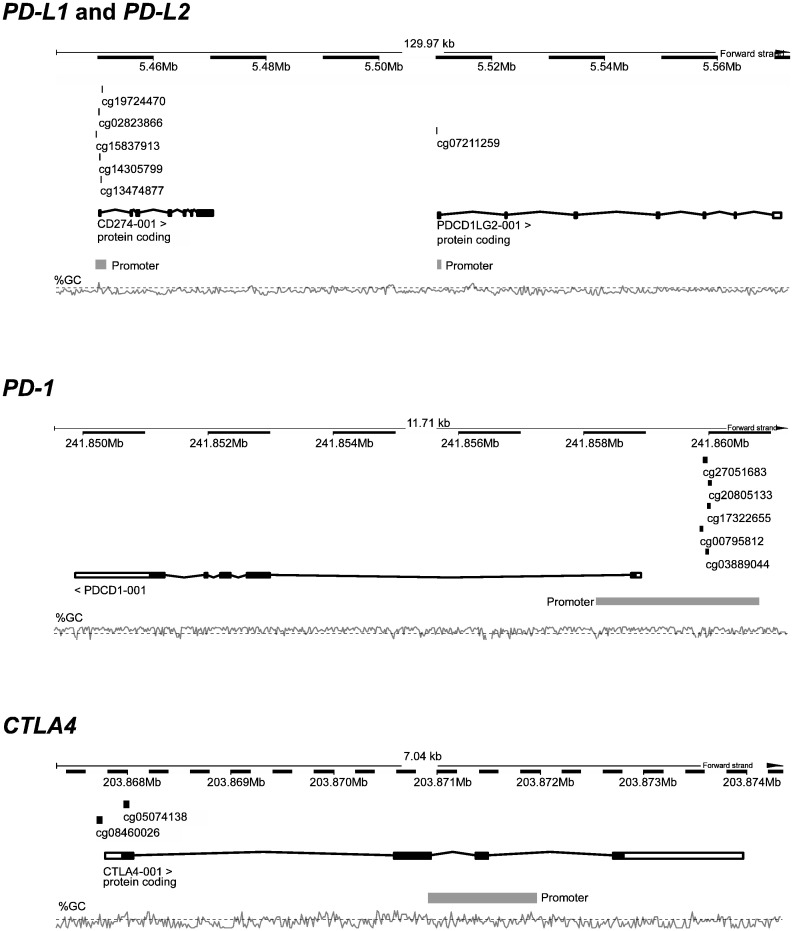
Genomic location and organization of *CD274* (*PD-L1*) and *PDCD1LG2* (*PD-L2*) on chromosome 9, *PDCD1* (*PD-1*) on chromosome 2, and *CTLA4* on chromosome 2. Analyzed cg-beads from the Illumina Infinium HumanMethylation450 BeadChip are illustrated. Figure information is based on the Genome Reference Consortium Human Build 38 patch release 7 (GRCh38.p7) illustrated by http://www.ensembl.org.

TCGA methylation data were generated using the Infinium HumanMethylation450 BeadChip (Illumina, Inc., San Diego, CA, USA). Relative DNA methylation levels were calculated as previously described for each locus (Meller et al., 2016). In brief, HumanMethylation450 data of level 2 including background-corrected methylated (Intensity_M) and unmethylated (Intensity_U) summary intensities (beads cg15837913, cg02823866, cg14305799, cg13474877, cg19724470 [*PD-L1*]; cg07211259 [*PD-L2*]; cg20805133, cg00795812, cg27051683, cg17322655, cg03889044 [*PD-1*]; cg05074138 and cg08460026 [*CTLA4*]) were downloaded and extracted by the R package ‘methylumi’. Methylation values for each bead were calculated with the formula: methylation [%] = 100% × Intensity_M / (Intensity_M + Intensity_U). Results from all beads from one gene were mean averaged. The genomic organization of the genes and the target regions of the analyzed beads are shown in Fig. 1.

### 2.3 mRNA Expression Analyses

mRNA data generated by the TCGA Research Network using the Illumina HiSeq 2000 RNA Sequencing Version 2 analysis (Illumina, Inc., San Diego, CA, USA) were obtained from the TCGA webpage and included normalized gene expression results. Counts per gene were calculated with the RSEM algorithm using the SeqWare framework (Li and Dewey, 2011).

### 2.4 Statistical Analyses

Statistical analyses were performed using SPSS, version 23.0 (SPSS Inc., Chicago, IL). Mean values are given ± standard deviation. Comparisons of mean values between groups were performed applying one way ANOVA with Bonferroni post-hoc testing, the Wilcoxon-Mann-Whitney test, and the Kruskal-Wallis test. Spearman's ρ rank correlations between mRNA and methylation levels were performed. Survival was defined as OS. Hazard ratios (HR) were calculated using univariate and multivariate Cox proportional hazards models with stratification. For survival analyses, continuous methylation data were logarithmized to base 2. Survival analyses were performed using the Kaplan-Meier method, and differences between the groups were tested using the log-rank test. For Kaplan-Meier survival analysis, methylation data were dichotomized using the median methylation level as cut-off. *p*-Values < 0.05 were considered as statistically significant.

## 3 Results

### 3.1 Correlation of *PD-1*, *PD-L1*, *PD-L2*, and *CTLA4* DNA Promoter Methylation With mRNA Expression

The Infinium HumanMethylation450 BeadChip contains 27 beads targeting the *PD-1* gene locus (Chr2:241849445–241860885; Reference Consortium Human Build 38 patch release 7 (GRCh38.p7)), 10 beads in the region of the adjacent genes *PD-L1* and *PD-L2* (Chr9:5445953–5572886), and seven beads probing the *CTLA4* gene locus (Chr2:203865714–203874906). In order to avoid multiple testing errors, we have reduced the analyses to pre-specified loci identified in previous studies (Fig. 1) (Franzen et al., 2018, Gevensleben et al., 2016, Goltz et al., 2016a, Goltz et al., 2016b, Goltz et al., 2017a, Goltz et al., 2017b). We found a significant inverse correlation between gene methylation and mRNA expression levels for PD-L1, PD-L2 and CTLA-4 (PD-L1: ρ = − 0.136, *p* = 0.005; PD-L2: ρ = − 0.642, *p* < 0.001; CTLA-4: ρ = − 0.249, *p* < 0.001), while no correlation was present for PD-1 (ρ = 0.020, *p* = 0.68).

### 3.2 Association of *PD-1*, *PD-L1*, *PD-L2*, and *CTLA4* DNA Promoter Methylation With Clinicopathological Features and Molecular Targets

For a detailed association analysis of promoter methylation of immune checkpoints with clinicopathological characteristics and molecular targets see Table 1. In brief, immune checkpoint promoter methylation was significantly associated with patients' age (*PD-L2*: *p* = 0.003; *PD-1*: *p* = 0.015), *O*^*6*^*-methylguanine DNA methyltransferase*(*MGMT*) methylation (*PD-L1*: *p* < 0.001; *PD-L2*: *p* < 0.001), *ATRX* mutations (*PD-L2*: *p* < 0.001, *PD-1*: *p* = 0.001), telomerase reverse transcriptase (*TERT*) mutations (*PD-L1*: *p* = 0.035, *PD-L2*: *p* < 0.001, *PD-1*: *p* < 0.001, *CTLA4*: *p* < 0.001), and methylation subgroups (LGm1, LGm2 and LGm3; *PD-L1*: *p* < 0.001, *PD-L2*: *p* < 0.001, *PD-1*: *p* = 0.001, *CTLA4*: *p* < 0.001).

### 3.3 *PD-1*, *PD-L1*, *PD-L2*, and *CTLA4* DNA Promoter Methylation in Glioma Methylation Subgroups

In a pan-glioma unsupervised cluster analysis, Ceccarelli et al. (2016) previously identified specific methylation subtypes LGm1/LGm2/LGm3, which carried *IDH1* or *IDH2* mutations, were enriched for lower-grade gliomas, and presented with a genome-wide hypermethylation compared to other methylation clusters. As our association analysis had already revealed a significant association with these methylation clusters (Table 1), we further analyzed the relationship of immune checkpoint methylation with methylation subtypes.

**Fig. 2:**
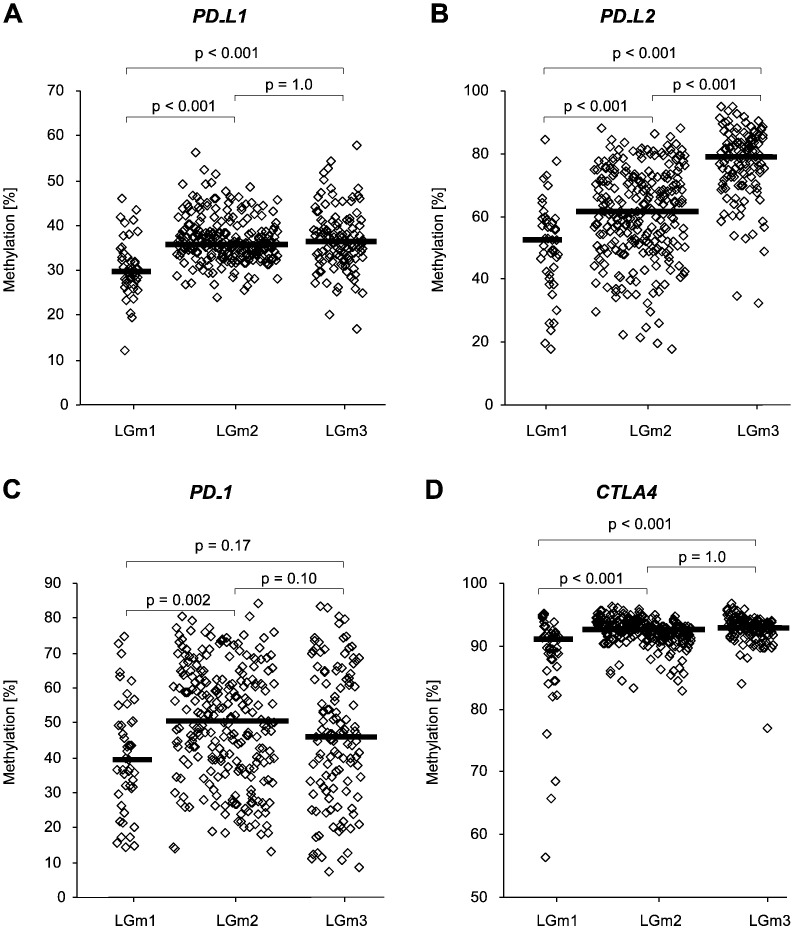
Methylation of immune checkpoint genes *PD-L1* (A), *PD-L2* (B), *PD-1* (C), and *CTLA4* (D) in diffuse lower-grade glioma patients (*n* = 419) with respect to methylation subtypes (LGm1, LGm2, LGm3). Bars indicate median. *p*-Values refer to ANOVA with Bonferroni Post hoc test.

Mean promoter methylation of both *PD-L1* and *PD-L2* was significantly lower in LGm1 (*PD-L1*: 30.6% ± 6.4%; *PD-L2*: 50.6% ± 1.5%) compared to LGm2 and LGm3 (*PD-L1*: 36.7% ± 5.0%, *p* < 0.001 for LGm1 vs. LGm2; 36.9% ± 6.6%, *p* < 0.001 for LGm1 vs. LGm3, Fig. 2A; *PD-L2*: 60.8% ± 14.5%, *p* < 0.001 for LGm1 vs. LGm2; 77.1% ± 11.2%, *p* < 0.001 for LGm1 vs. LGm3; Fig. 2B). Additionally, *PD-L2* levels were shown to be lower in LGm2 compared to LGm3 (*p* < 0.001). Mean *PD-1* promoter methylation was significantly lower in LGm1 (40.5% ± 16.7%) compared to LGm2 (50.1% ± 16.4%, *p* = 0.002; Fig. 2C). No significant differential promoter methylation was seen for LGm3 (46.1% ± 19.2%). Mean *CTLA4* promoter methylation was significantly lower in LGm1 (88.2% ± 7.8%) compared to both LGm2 and LGm3 (92.3% ± 22.2%, *p* < 0.001 for LGm1 vs. LGm2; 92.7% ± 2.3%, *p* < 0.001 for LGm1 vs. LGm3; Fig. 2D). Of note, the heterogeneity of values was shown to be significantly higher for *PD-1* and *CTLA4* methylation possibly indicating that methylation levels do not reflect the rather homogenous tumor tissue but might be distorted by PD-1 and CTLA-4 expressing infiltrating immune cells.

### 3.4 Correlation of *PD-1*, *PD-L1*, *PD-L2*, and *CTLA4* DNA Promoter Methylation With Immune Cell Infiltrates

Since PD-1 and CTLA-4 expression has been mainly observed in immune cells (Buchbinder and Desai, 2016), differential *PD-1* and *CTLA4* promoter methylation may reflect changes in the lymphocyte and antigen presenting cell compartment. Subtypes of tumor infiltrating lymphocytes in the TCGA cohort as assessed by Li et al. (2016) were correlated with *PD-1* and *CTLA4* promoter methylation. Tumor infiltrating B lymphocytes as well as CD8 positive (CD8^+^) T lymphocytes and dendritic cells correlated inversely with *PD-1* (*r* = − 0.178; *p* < 0.001 for B lymphocytes, *r* = − 0.234; *p* < 0.001 for CD8^+^ T lymphocytes, and *r* = − 0.171; *p* < 0.031 for dendritic cells, *n* = 419 for all). For *CTLA4* methylation, no significant association with immune cells was observed. Promoter methylation of *PD-L1* significantly correlated with *PD-1* methylation in tumor samples (*r* = 0.293; *p* < 0.001; *n* = 419). Further, *PD-L1* methylation significantly and inversely correlated with infiltrating CD4 positive (CD4^+^) T lymphocytes (*r* = − 0.109; *p* = 0.026; *n* = 419) and dendritic cells (*r* = − 0.099; *p* = 0.043; *n* = 419). Promoter methylation of *PD-L2* significantly correlated with *PD-1* methylation in tumor samples (*r* = 0.186; *p* < 0.001; *n* = 419). However, no association was found with immune cells.

### 3.5 Prognostic Impact of *PD-1*, *PD-L1*, *PD-L2*, and *CTLA4* DNA Promoter Methylation

**Fig. 3:**
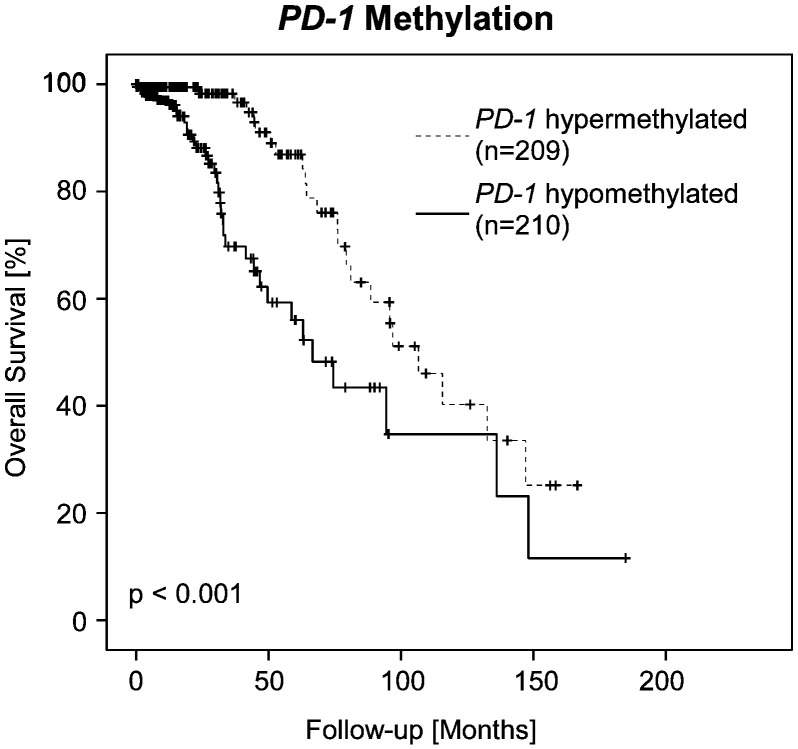
Kaplan-Meier analysis of overall survival in diffuse lower-grade glioma patients (*n* = 419) stratified by promoter methylation of *PD-1*.

Subsequently, we analyzed whether promoter methylation of immune checkpoints allowed for a risk stratification of LGG patients. Since the DNA methylation clusters had been shown to have an impact on survival, Cox proportional hazard analysis was stratified according to methylation subtypes (LGm1, LGm2 and LGm3). In univariate and multivariate Cox proportional hazard analysis, *PD-1* methylation qualified as a strong prognostic factor together with age (univariate Cox proportional hazard analysis: HR = 0.44 [0.30–0.66], *p* < 0.001; multivariate Cox proportional hazard analysis: HR = 0.51 [0.34–0.76], *p* = 0.001; Table 2). The prognostic value of dichotomized *PD-1* methylation was further confirmed by Kaplan-Meier analysis (Χ^2^ = 13.04, *p* < 0.001 for hypomethylated *PD-1* and hypermethylated *PD-1*, respectively; Fig. 3). No prognostic impact was observed for *PD-L1*, *PD-L2*, and *CTLA4* methylation.

**Table 2:** Univariate and multivariate Cox proportional hazard analysis of immune checkpoint methylation, sex, and age. Methylation was analyzed as logarithmized continuous variable. Cox analysis was stratified by methylation subtype (LGm1, LGm2 and LGm3)^a^.

Variable	Univariate cox proportional hazards analysis		Multivariate cox proportional hazards analysis	
	*p*	HR [95% CI]	*p*	HR [95% CI]
Age at initial diagnosis (> 41 vs. ≤ 41 years)	< 0.001	3.83 [2.04–7.17]	< 0.001	3.19 [1.69–6.02]
Sex (male vs. female)	0.62	1.15 [0.67–1.96]	0.75	1.09 [0.64–1.87]
*PD-1* methylation	< 0.001	0.44 [0.30–0.66]	0.001	0.51 [0.34–0.76]
*PD-L1* methylation	0.25	0.57 [0.22–1.48]	ND	
*PD-L2* methylation	0.16	0.63 [0.33–1.20]	ND	
*CTLA4* methylation	0.26	0.17 [0.01–3.69]	ND	

a Data taken from Ceccarelli et al. (2016); ND: not determined.

## 4 Discussion

In the present study, DNA promoter methylation of the immune checkpoint *PD-1* was shown to serve as highly significant prognostic factor for overall survival in patients with LGG. In its key role as an immune checkpoint PD-1 promotes self-tolerance by suppressing T cell activity. Upon binding to its ligands PD-L1 or PD-L2, PD-1 fosters apoptosis in antigen specific T cells while simultaneously reducing programmed cell death in regulatory T cells (Tregs) (Francisco et al., 2009, Francisco et al., 2010). PD-1 is mainly expressed on activated CD4^+^ and CD8^+^ T cells as well as on B cells and inhibits effector T cell activity at later-stage immune responses in peripheral tissues (Francisco et al., 2010). The interaction of PD-L1 on tumor cells with PD-1 on tumor-specific T cells has been identified as an important mechanism of immune evasion of tumor cells with high PD-1 expression consequently leading to depleted antitumor immune responses (reviewed by Zhang et al., 2017). Recent publications have further provided evidence for an epigenetic promoter control of PD-1 expression in human T lymphocytes (Youngblood et al., 2011, McPherson et al., 2014).

As gliomas have long been recognized as immunosuppressive neoplasms that are characterized by the activation of various immune escape mechanisms (reviewed by Razavi et al., 2016), our findings indicate that high levels of *PD-1* promoter methylation might result in functional tumor-specific T cells effectively driving antitumor immune responses, therefore leading to a favorable course of disease. In addition, promoter methylation of *PD-L1* also significantly correlated with *PD-1* methylation, suggesting that epigenetic regulation of the *PD-1* receptor may be paralleled by PD-L1 induction in tumor tissue. *PD-1* methylation further inversely correlated with tumor infiltrating B lymphocytes, CD8^+^ T lymphocytes, and antigen presenting dendritic cells in our study, suggesting a role of *PD-1* methylation as surrogate marker for immune cell infiltration. In addition, *PD-L1* methylation inversely correlated with infiltrating CD4^+^ T lymphocytes and dendritic cells, adding to the seemingly reciprocal relationship of infiltrating immune cells and immune checkpoint methylation in LGG.

Tumor-infiltrating immune cells are part of a complex microenvironment that is known to regulate tumor development and growth in gliomas (reviewed by Domingues et al., 2016); however, data on the role of immune cells and their influence on survival have been conflicting. While several studies have demonstrated that high numbers of intratumoral effector T cells are significantly correlated with a better survival in grade IV gliomas (Lohr et al., 2011, Kmiecik et al., 2014), other groups have reported that specific molecular subtypes (e.g. the mesenchymal subtype) are characterized by pro-inflammatory immune signatures and immunosuppression, associating immune infiltrates to a higher risk and poor survival (Doucette et al., 2013, Cheng et al., 2016). Of note, neither tumor infiltrating lymphocytes, nor antigen presenting cells added prognostic information in our study (data not shown). Although further mechanistic studies are clearly warranted in order to fully characterize the role of PD-1 expression in LGG, our results imply that the densities of B and CD8^+^ T lymphocytic infiltrates as well as antigen presenting dendritic cells might be estimated via *PD-1* methylation. This might be of significance for the potential therapeutic application of immunotherapies in LGG patients in the future.

A publication by Ceccarelli et al. (2016) has recently emphasized the relevance of DNA methylation for the clinical classification and biological behavior of gliomas. Unsupervised cluster analysis of 1122 diffuse glioma patients identified six DNA methylation subtypes with distinct molecular and clinical features. In this study, the LGm1/LGm2/LGm3 subgroups harbored *IDH1* or *IDH2* mutations (449 of 450, 99%), were enriched for LGG (421/454, 93%), and showed genome-wide hypermethylation compared to LGm4–6 clusters, corroborating the association between *IDH* mutation and increased DNA methylation (Turcan et al., 2012). Interestingly, further analysis between the two discovered *IDH* mutant-non-codel DNA methylation clusters allowed for the identification of a low-methylation subgroup (G-CIMP-low) which was enriched in the heterogeneous subgroup of LGm1 tumors. As *PD-1*, *PD-L1*, *PD-L2*, and *CTLA4* promoter methylation was significantly lower in the LGm1 subgroup compared to LGm2 and LGm3 in the present study, our results might therefore very well reflect the hypermethylated phenotype in LGm2 and LGm3 as opposed to a low-methylated subgroup enriched in LGm1. In line with previous results, Ceccarelli et al. (2016) also reported the G-CIMP-low subgroup to be associated with a worse survival compared to hypermethylated tumors. In our study, methylation of *PD-L2* additionally distinguished between LGm2 and LGm3, thus suggesting differential methylation between these two subgroups independent from cluster-related hypermethylation.

Some of the differences in mean methylation found in our study were remarkably small. However, a strength of our study is the large number of included patients allowing for the detection of even small differences with high statistical significance. The biological significance appears to be supported by the finding of a strong inverse correlation between mRNA expression and methylation levels for *PD-L2*, *CTLA4*, and *PD-L1*. *CTLA4* methylation in *TERT*-mutated versus wildtype tumors, for example, seems minor at first glance (93.22% versus 92.45% methylation). However, this difference is equivalent to 6.78% (100%–93.22%) versus 7.55% (100%–92.45%) “unmethylation” which represents a remarkable increase of “unmethylation” by 11%. These findings indicate that a small subgroup of (unmethylated) cells is responsible for the mRNA expression. A major limitation of our study, however, is that we were not able assign methylation and expression levels to specific cell types. This needs to be done in further studies. A second limitation of our study is the relatively short follow-up. LGG patients harboring an *IDH* mutation and 1p/19q codeletion for example, representing 40.3% of the cohort under investigation, have a relatively good prognosis with a median overall survival of 8 years (Cancer Genome Atlas Research Network et al., 2015). Hence, long follow-up periods are required in order to detect statistically significant survival differences. The high prognostic power of *PD-1* promoter methylation presented in our study, even in the absence of long follow-up, however, indicates the high relevance of this gene in LGG. The lack of prognostic power of *PD-L1*, *PD-L2*, and *CTLA4* methylation, on the other hand, might be due to the limited follow-up, and the prognostic potential of these markers might need to be investigated in a study with sufficient clinical follow-up. The lack of a validation study in our analysis further prompted us to analyze only CpG-sites which have been shown to be of significance in earlier studies (Franzen et al., 2018, Gevensleben et al., 2016, Goltz et al., 2016a, Goltz et al., 2016b, Goltz et al., 2017a, Goltz et al., 2017b). As previously reported, we used mean methylation values of these CpG-sites. Such approach reduces the risk of multiple testing errors since only a limited number of (predefined) features is analyzed. On the other hand, the prognostic performance might be underestimated since other or single individual CpG-sites might be more informative than the predefined and averaged ones. This needs to be addressed in further studies.

Inhibition of the PD-1/PD-L1 and CTLA-4 immune checkpoints is a promising therapeutic approach for the treatment of primary brain tumors. Several clinical trials are currently ongoing and evaluating the effects of immune checkpoint inhibition in high-grade glioma with nivolumab, pembrolizumab and ipilimumab. First study results, however, so far have not painted a clear picture. While the PD-1 checkpoint inhibitor nivolumab recently failed to demonstrate a survival benefit in a first randomized clinical phase III trial (CheckMate 143, ClinicalTrials.gov Identifier: NCT02017717) in high-grade glioma, pembrolizumab appears to have a durable benefit for patients with recurrent PD-L1-positive glioblastomas (Omuro et al., 2017, Reardon et al., 2016). In the context of rapidly developing immunotherapies, e.g. checkpoint inhibitors, robust and clinically applicable biomarkers are needed to estimate the potential effects and identify patients eligible for treatment. In the present study, DNA methylation analysis of *PD-1* in LGG patients was shown to add independent prognostic information. Furthermore, DNA methylation analysis can be conducted reliably even in small and formalin-fixed samples. We therefore strongly recommend the integration of immune checkpoint promoter methylation analysis in running and future clinical trials in order to test its ability to predict treatment response to immune checkpoint inhibitors.

## Funding Sources

No funding was received.

## Conflicts of Interest

Dimo Dietrich is inventor and owns a granted patent on methylation of immune checkpoint genes as prognostic and predictive biomarkers (patent application DE102016005947 (B3)). A second patent application on immune checkpoint genes as biomarkers for immunotherapy response prediction is pending.

## Author Contributions

LKR analyzed the data and drafted the manuscript. HG and DG coordinated the study, analyzed the data and drafted the manuscript. JD processed TCGA data. FB and JL revised the manuscript critically for important intellectual content. DD designed and supervised the study, analyzed the data and revised the manuscript.
